# Comparison of logistic regression and machine learning methods for predicting postoperative delirium in elderly patients: A retrospective study

**DOI:** 10.1111/cns.13991

**Published:** 2022-10-11

**Authors:** Yu‐xiang Song, Xiao‐dong Yang, Yun‐gen Luo, Chun‐lei Ouyang, Yao Yu, Yu‐long Ma, Hao Li, Jing‐sheng Lou, Yan‐hong Liu, Yi‐qiang Chen, Jiang‐bei Cao, Wei‐dong Mi

**Affiliations:** ^1^ Department of Anesthesiology The First Medical Center of Chinese PLA General Hospital Beijing China; ^2^ Medical School of Chinese People's Liberation Army Beijing China; ^3^ Institute of Computing Technology Chinese Academy of Sciences Beijing China

**Keywords:** aged, delirium, machine learning, nomograms, risk assessment

## Abstract

**Aims:**

To compare the performance of logistic regression and machine learning methods in predicting postoperative delirium (POD) in elderly patients.

**Method:**

This was a retrospective study of perioperative medical data from patients undergoing non‐cardiac and non‐neurology surgery over 65 years old from January 2014 to August 2019. Forty‐six perioperative variables were used to predict POD. A traditional logistic regression and five machine learning models (Random Forest, GBM, AdaBoost, XGBoost, and a stacking ensemble model) were compared by the area under the receiver operating characteristic curve (AUC‐ROC), sensitivity, specificity, and precision.

**Results:**

In total, 29,756 patients were enrolled, and the incidence of POD was 3.22% after variable screening. AUCs were 0.783 (0.765–0.8) for the logistic regression method, 0.78 for random forest, 0.76 for GBM, 0.74 for AdaBoost, 0.73 for XGBoost, and 0.77 for the stacking ensemble model. The respective sensitivities for the 6 aforementioned models were 74.2%, 72.2%, 76.8%, 63.6%, 71.6%, and 67.4%. The respective specificities for the 6 aforementioned models were 70.7%, 99.8%, 96.5%, 98.8%, 96.5%, and 96.1%. The respective precision values for the 6 aforementioned models were 7.8%, 52.3%, 55.6%, 57%, 54.5%, and 56.4%.

**Conclusions:**

The optimal application of the logistic regression model could provide quick and convenient POD risk identification to help improve the perioperative management of surgical patients because of its better sensitivity, fewer variables, and easier interpretability than the machine learning model.

## Graphical abstract

Six prediction models were constructed for POD using logistic regression, RF, AdaBoost, XGBoost, GBM, and stacking ensemble learning based on retrospective analysis of a large sample dataset. The logistic regression model had the same AUC(0.78) with the RF, and performed better than the machine learning models because of its better sensitivity, fewer variables, and easier interpretability.

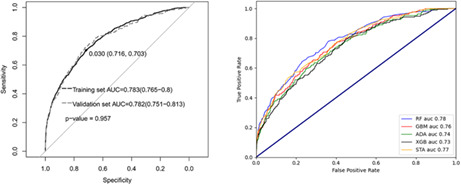

## 1 INTRODUCTION

Delirium is an acute clinically reversible syndrome characterized by a typical dysfunction of cognition and attention after anesthesia and surgery^1^. Postoperative delirium (POD) commonly occurs 2–7 days postoperatively^2^. Evidence indicates that POD is a common and severe complication in patients undergoing major surgery^1^, ^3^, ^4^. Although its incidence in the general surgical population is 2%–3%, it has been reported in up to 50%–70% of high‐risk patient groups^5^. Adverse effects of POD are loss of independence, increased morbidity and mortality, institutionalization, and high healthcare costs^6^, ^7^. Studies have shown that one‐third of delirium cases can benefit from multifactorial preventive measures and treatments^8^. Therefore, it is critical to immediately identify high‐risk patients postoperatively, as this can help clinicians improve the outcome of patients by timely intervention. The etiology of POD was not clear. Low neuronal metabolism and dysfunctional cerebral autoregulation may associate with POD^9^, ^10^. It is difficult to predict the POD in terms of etiology. So, most researchers predicted POD by preoperative or/and intraoperative clinical parameters.

Machine learning is a type of artificial intelligence that allows software applications to become more accurate at predicting outcomes without being explicitly programmed to do so. As a result of machine learning models' ability to learn from multiple modules of data and their robustness to data noise, localized specific predictions can be made. Furthermore, machine learning may be able to analyze the underlying mechanisms of a variety of complications^11^. Some studies reported that the urinary albumin creatinine ratio and systemic immune‐inflammation index (SII) had the prediction value for POD^12^, ^13^. But the AUC (area under the ROC curve) of them did not perform well. Besides, some models have been developed to predict delirium using logistic regression or machine learning methods for different surgeries^14^, ^15^, ^16^, ^17^. A nomogram model with logistic regression was usually used to predict POD in a particular type of surgery in small‐scale cohorts^16^, ^17^. Machine learning methods can be optimally applied when data are abundant^18^, and they have been widely used in various clinical domains to predict events of interest^15^, ^19^, ^20^. Nevertheless, it remains controversial whether complex machine learning algorithms can surpass conventional generalized linear models in specific areas^21^. Moreover, use of the model has been limited, even though such methods have proven to be efficient. These predictions relied on statistical features instead of clinically meaningful variables for the non‐paraphrasing of machine learning.

Therefore, we compared the performance of logistic regression and machine learning methods in predicting POD to develop a clinically meaningful model to support clinical decision‐making.

## 2 MATERIALS AND METHODS

### 2.1 Ethics statements

The study was conducted in accordance with the Declaration of Helsinki and approved by the Ethics Committee Board of the First Medical Center of the Chinese PLA General Hospital (number: S2019‐311‐03). The need for patient consent was waived due to the retrospective study design and all data were anonymized before analysis.

### 2.2 Study design and patients

In this retrospective cohort study, a dataset of patients who underwent surgery at the First Medical Center of the Chinese PLA General Hospital from January 2014 to April 2019 was analyzed. The inclusion criteria were as follows: (1) age ≥ 65 years and (2) patients undergoing surgery with anesthesia. The exclusion criteria were as follows: (1) patients undergoing neurosurgery or cardiac surgery, (2) patients undergoing digestive endoscopy, and (3) patients with >50% of data missing.

### 2.3 Data collection

The dataset was established using a medical record system. For more accurate and wider clinical use of the models, we included the following preoperative and intraoperative parameters that might be closely associated with POD in the models.

1. We collected relevant patient demographics, including age, sex, body mass index (BMI), combined hypertension, diabetes, cardiovascular diseases, chronic obstructive pulmonary disease, chronic kidney disease, Parkinson's disease, cerebrovascular disease, depression, non‐independent functional status, and American Society of Anesthesiologists (ASA) grade.
2. We recorded the prescribed medication before surgery: non‐steroidal anti‐inflammatory drugs (loxoprofen, acetaminophen, ibuprofen, celecoxib, meloxicam, diclofenac, etoricoxib, and nimesulide), anticholinergic drugs (atropine, penehyclidine hydrochloride, and scopolamine), benzodiazepines (midazolam, estazolam, diazepam, lorazepam, alprazolam, zolpidem, and zopiclone), opioids (tramadol, oxycodone, and fentanyl transdermal patch), and antipsychotic drugs (quetiapine, olanzapine, droperidol, haloperidol, and risperidone).
3. The following laboratory test results (the last time before surgery) were collected: levels of hemoglobin, white blood cell (WBC) count, glucose (Glu), serum albumin, erythrocyte sedimentation rate, serum creatinine (Cre), blood potassium, blood sodium, blood calcium, alanine aminotransferase, and aspartate aminotransferase.
4. The following intraoperative data were recorded: the type of surgery, anesthesia method, emergency surgery, duration of surgery and anesthesia, urine output, blood loss, use of dexmedetomidine, use of droperidol, crystalloid fluid management, colloid fluid management, blood transfusion (red blood cells, whole blood, plasma, platelets, cryoprecipitate, autologous blood, fibrinogen, and albumin), use of glucocorticoids (dexamethasone and methylprednisolone), duration of systolic blood pressure > 140 mmHg, and duration of mean arterial pressure < 60 mmHg.

### 2.4 Definitions of outcomes

The primary outcome was the incidence of POD within 7 days postoperatively. First, the data were captured using descriptive words documented in the medical records. The inclusion criteria were as follows: (1) the postoperative medical records contained “mental status change,” “confusion,” “disorientation,” “agitation,” “delirium,” “inappropriate behavior,” “inattention,” “hallucinations,” “combative behavior,” “drowsy,” “slept poorly,” and other similar meaning words in Chinese^22^, ^23^; and (2) the postoperative drug manuscript contained “quetiapine,” “olanzapine,” “haloperidol,” “haloperidol,” and “risperidone.” Exclusion criteria were as follows: (1) the preoperative medical records contained the aforementioned “symptoms;” and (2) the preoperative drug manuscript contained the aforementioned “drugs.”. Second, the patients preliminarily diagnosed by a computer were rechecked by neurologists using the Diagnostic and Statistical Manual of Mental Disorders, fourth edition (DSM‐IV) criteria^24^.

### 2.5 Model building strategy

A logistic regression model was used to predict POD outcomes. Patients were randomly split into training and validation datasets at a ratio of 3:1. Variables showing statistical significance in the univariate analysis were included in the multivariable logistic regression analysis, and the forward and backward stepwise methods were used to select the variables that were eventually included in the model. The equation of logistic regression was displayed as the follow:$P=\frac{1}{1+e^{-g\left(x\right)}}$, $g_{\left(x\right)}=\omega_{0}+\omega_{1}x_{1}+\ldots +\omega_{n}x_{n}$. Based on the regression coefficients of the independent variables, we established an individualized nomogram prediction model for POD in major surgery. The prediction model was evaluated in patients from the validation cohort. The discrimination ability of the prediction model was assessed by calculating the area under the receiver operating characteristic curve (AUC). The calibration of the model was evaluated using the Hosmer–Lemeshow goodness‐of‐fit test. Decision curve analysis (DCA) revealed the net benefits for each threshold probability.^25^

Machine learning models were developed with four different types of models: random forest (RF), gradient boosting machine (GBM), adaptive boosting with classification trees (AdaBoost), and extreme gradient boosting with classification trees (XGBoost). Subsequently, a stacking model was constructed using the four aforementioned machine learning models. The patients were randomly split into two datasets with split ratios of 80% and 20%. Subsequently, 20% of the patients were used for testing. Eighty percent of the patients were used for training.

As aforementioned, the incidence of POD in the general surgical population is 2%–3% and leads to an imbalanced dataset where positive patients are much fewer than the negative ones, which leads to the learned model would be biased toward the negative category, that is, non‐POD. Resampling is an effective way to mitigate the imbalance distribution influence, among which oversampling the minority class has been proven to be better than undersampling the majority class because of losing no information. To avoid the overfitting caused by oversampling, the synthetic minority oversampling technique (SMOTE)^26^, ^27^was used to synthetically generate positive samples instead of simply duplicating them. Furthermore, SMOTE chose those positive samples that were near the negative category as the synthetic basis, which makes the oversampling more effective and robust, that was also called borderline SMOTE^28^.

Hyperparameter tuning was performed using five‐fold cross‐validation. Specifically, the derivation dataset was divided into five subsets and the holdout method was repeated five times. One of the five subsets was used as the validation set, and the other four subsets were combined to form a derivation set. The average AUC across all five trials was calculated. The hyperparameters were tuned using a grid search for each algorithm. The combination of hyperparameters with the highest AUC was used for model development. Model stacking, a method used to improve model predictions by combining the outputs of multiple models and running them through another machine learning model called a meta‐learner^29^, was developed by applying RF, GBM, AdaBoost, and XGBoost. Essentially, a stacked model works by running the output of multiple models through a “meta‐learner” that attempts to minimize the weakness and maximize the strengths of every individual model. The result is typically a robust model that generalizes well on unseen data. We used RF as the base model and the other three mode is as the meta‐models. The importance and correlation of the variables have been reported to facilitate model interpretation.

### 2.6 Statistical analysis

Kolmogorov–Smirnov test was used to assess data distribution. Normally distributed continuous variables are expressed as mean (standard deviation) and were compared using Student's t‐test. If continuous data were not normally distributed, they are shown as the median and interquartile range and were compared using a non‐parametric equivalent (Mann–Whitney's test). Categorical variables are expressed as frequency or percentage and were compared using the χ^2^ test or Fisher's exact test. The interaction test was performed for the variables in the logistic regression model.

Statistical significance was accepted at a level of 0.05, and all tests were two‐tailed. The logistic regression model was performed using R 4.0.1 (R Foundation for Statistical Computing). Machine learning models were developed using PyCharm 11.0.14.1 (JetBrains s.r.o.,).

## 3 RESULTS

### 3.1 Patient characteristics

The medical records of 31,363 patients who were older than 65 years of age and underwent non‐cardiac and non‐neurological surgery from January 2014 to August 2019 at the First Medical Center of Chinese PLA General Hospital were retrospectively analyzed. We excluded 1241 patients who underwent digestive endoscopy and 491 patients because of missing data. Finally, 29,756 patients were included in the following analysis (Figure S1). The patient characteristics and perioperative variables for the overall population are shown in Table 1. Overall, 961 patients (3.23%) developed POD within 7 days postoperatively. We enrolled all patients undergoing surgery, including ophthalmic, ear‐nose‐throat, and oral surgery with a low POD incidence and excluded cardiac surgery and neurosurgery with a high POD incidence. Therefore, the 3.2% incidence of POD in our study is reasonable. Patients' median (interquartile range) age was 70 (67, 74) years, and 14,606 (49.1%) patients were male. The median (interquartile range) duration of surgery was 145 (103, 205) minutes, and the median (interquartile range) duration of anesthesia was 195 (150, 258) minutes. The patients suffering from POD were significantly older than patients without POD [73 (68–78) vs 70 (67–74) years, *p* < 0.001]. The duration of surgery and anesthesia were also significantly longer in patients with POD (Table 1). Table 2 shows the results of preoperative laboratory testing and perioperative medication. The patients with POD had significantly lower hemoglobin, serum albumin and higher WBC, Glu, and Cre. Before the surgery, more the POD patients used the opioids and antipsychotic drugs. The usage of glucocorticoid, dexmedetomidine, and Droperidol during the surgery were no difference between the two groups (Table 2).

**TABLE 1:** Patient characteristics and baseline variables

Characteristics	Non‐POD(*n* = 28,795)	POD (*n* = 961)	*p*‐value
Age, years	70(67–74)	73(68–78)	<0.001
Sex (male), *n* (%)	14,195(49.3)	411(42.8)	<0.001
BMI, kg·m^2^	24.49(22.27–26.89)	23.88(21.22–26.13)	<0.001
Smoke, *n* (%)	6659(23.1)	262(27.3)	0.003
Alcohol, *n* (%)	6217(21.6)	228(23.7)	0.123
Hypertension, *n* (%)	13,808(48)	502(52.2)	0.01
Diabetes, *n* (%)	6770(23.5)	265(27.6)	0.004
Cardiovascular diseases, *n* (%)	2786(9.7)	123(12.8)	0.002
COPD, *n* (%)	1117(3.9)	90(9.4)	<0.001
Cerebrovascular disease, *n* (%)	2801(9.7)	143(14.9)	<0.001
Parkinson's disease, *n* (%)	116(0.4)	12(1.2)	<0.001
CKD, *n* (%)	336(1.2)	36(3.7)	<0.001
Depression, *n* (%)	125(0.4)	13(1.4)	<0.001
Non‐independent functional status, *n* (%)	8389(29.1)	428(44.5)	<0.001
ASA, *n* (%)			<0.001
I	344(1.2)	7(0.7)	
II	22,958(79.7)	536(55.8)	
III	5306(18.4)	351(36.5)	
IV	151(0.5)	50(5.2)	
V	36(0.1)	17(1.8)	
Emergency surgery, *n* (%)	753(2.6)	112(11.7)	<0.001
Type of surgery, *n* (%)			<0.001
Hepatopancreatobiliary and gastrointestinal surgery	9883(34.3)	417(43.4)	
Orthopedic surgery	8462(29.4)	282(29.3)	
Urinary surgery	2599 (9)	64(6.7)	
Thoracic surgery	2128(7.4)	49(5.1)	
E.N.T	1541(5.4)	24(2.5)	
Vascular surgery	1198(4.2)	58(6)	
Stomatology	1191(4.1)	29(3)	
Gynecology	984(3.4)	24(2.5)	
Thyroid and Brest	809(2.8)	14(1.5)	
Anesthesia method, *n* (%)			0.058
General anesthesia	23,842(82.8)	807(84)	
General anesthesia combined with other anesthesia	3430(11.9)	111(11.6)	
Nerve blocks	601(2.1)	26(2.7)	
Epidural anesthesia	536(1.9)	7(0.7)	
Basal anesthesia	386(1.3)	10(1)	
Duration of surgery, log min	130(85–195)	166(114–240)	<0.001
Duration of anesthesia, min	180(130–245)	220(165–294)	<0.001
Blood loss, ml	100(30–200)	150(50–300)	<0.001
Urine, ml	200(100–500)	350(150–600)	<0.001
Crystalloid, ml	1400(1100–2100)	1600(1100–2200)	<0.001
Colloid, ml	1400(1100–2100)	1600(1100–2200)	<0.001
Blood transfusion, *n*	3964(13.8)	280(29.1)	<0.001
Duration of SBP > 140 mmHg, min	5(0–25)	15(0–40)	<0.001
Duration of MAP<60 mmHg, min	5(0–10)	5(0–20)	<0.001

*Note*: Data are mean (standard deviation), *n* (%), or median (interquartile range).

Abbreviations: ASA, American Society of Anesthesiologists physical status classification system; BMI, body mass index; CKD, chronic kidney disease; COPD, chronic obstructive pulmonary disease; E.N.T., otolaryngology head and neck surgery; MAP, mean arterial pressure; SBP, systolic blood pressure.

**TABLE 2:** The preoperative laboratory testing and perioperative medication

Variables	Non‐POD(*n* = 28,795)	POD (*n* = 961)	*p*‐value
Hemoglobin, g·L^−1^	130(119–141)	122(107–136)	<0.001
WBC count, *10^9^/L	5.92(4.94–7.14)	6.48(5.29–8.59)	<0.001
ESR, mm/h	0(0–3)	0(0–2)	0.808
Glu, mmol/L	5.12(4.66–5.9)	5.41(4.73–6.96)	<0.001
Serum albumin, g/L	39.9(37.3–42.3)	37.8(34.4–40.8)	<0.001
Cre, μmol/L	70.6(60.2–82.7)	73.4(61.3–88.9)	<0.001
Blood potassium, mmol/L	4.02(3.8–4.27)	4.04(3.79–4.3)	0.239
Blood sodium, mmol/L	142(140.1–143.6)	140.7(137.9–142.9)	<0.001
Blood calcium, mmol/L	2.23(2.17–2.3)	2.21(2.13–2.3)	<0.001
AST, U/L	14.7(10.9–21.8)	14.5(10–22)	0.023
ALT, U/L	16.8(14–21.3)	17.1(13.7–23)	0.323
Preoperative medication, *n* (%)			
Anticholinergic drug	16,314(56.7)	523(54.4)	0.18
NSAIDs	1985(6.9)	47(4.9)	0.018
Benzodiazepines	6390(22.2)	226(23.5)	0.351
Opioids	839(2.9)	74(7.7)	<0.001
Antipsychotic drugs	33(0.1)	74(7.7)	<0.001
Intraoperative medication, *n* (%)			
Glucocorticoid	17,993(62.5)	613(63.8)	0.432
Dexmedetomidine	2862(9.9)	104(10.8)	0.399
Droperidol	2594 (9)	71(7.4)	0.094

*Note*: Data are presented as mean (SD) or median (interquartile range) or number of patients (%).

Abbreviations: ALT, alanine aminotransferase; AST, aspartate aminotransferase; Cre, creatinine; ESR, erythrocyte sedimentation rate; Glu, glucose; NSAIDs, non‐steroidal anti‐inflammatory drugs; WBC, white blood cell.

### 3.2 Development and validation of a nomogram with the logistic regression model

A training dataset of 22,317 patients was used to develop the predictive model. The results of the univariate logistic regression analysis are shown in Table S1. Variables that were statistically significant in the univariate analysis were included in multivariate logistic regression analysis. Age, ASA grade, depression, emergency surgery, duration of anesthesia, WBC count, serum albumin level, and antipsychotic drugs were independent risk factors for POD (Table 3). The variance inflation factors of the independent risk factors were all <2 by collinearity diagnostics, suggesting no multicollinearity among the risk factors.

**TABLE 3:** Multivariable logistic regression model of study variables vs. POD in the training dataset

Variables	Odds Ratio (95%CI)	*p*‐value
Age, years	1.068(1.053–1.083)	<0.001
Depression, yes vs no	3.364(1.632–6.331)	<0.001
ASA, vs I‐II		
III	1.605(1.34–1.917)	0.373
IV‐V	5.235(3.441–7.85)	0.003
Emergency surgery, yes vs no	2.879(2.048–3.993)	<0.001
WBC count, *10^9^/L	2.06(1.643–2.585)	<0.001
Serum albumin, g/L	0.107(0.056–0.207)	<0.001
Antipsychotic drugs, yes vs no	47.139(27.997–81.056)	<0.001
Duration of anesthesia, min	2.498(1.993–3.139c)	<0.001

Abbreviations: ASA, American Society of Anesthesiologists physical status classification system; WBC, white blood cell.

In addition, the hemoglobin level, non‐independent functional status, Cre level, and blood loss were statistically significant in the univariate model and insignificant in the multivariable model. The use of anticholinergic drugs, non‐steroidal anti‐inflammatory drugs, and benzodiazepines showed no statistical significance in either the univariate or multivariate model.

**FIGURE 1:**
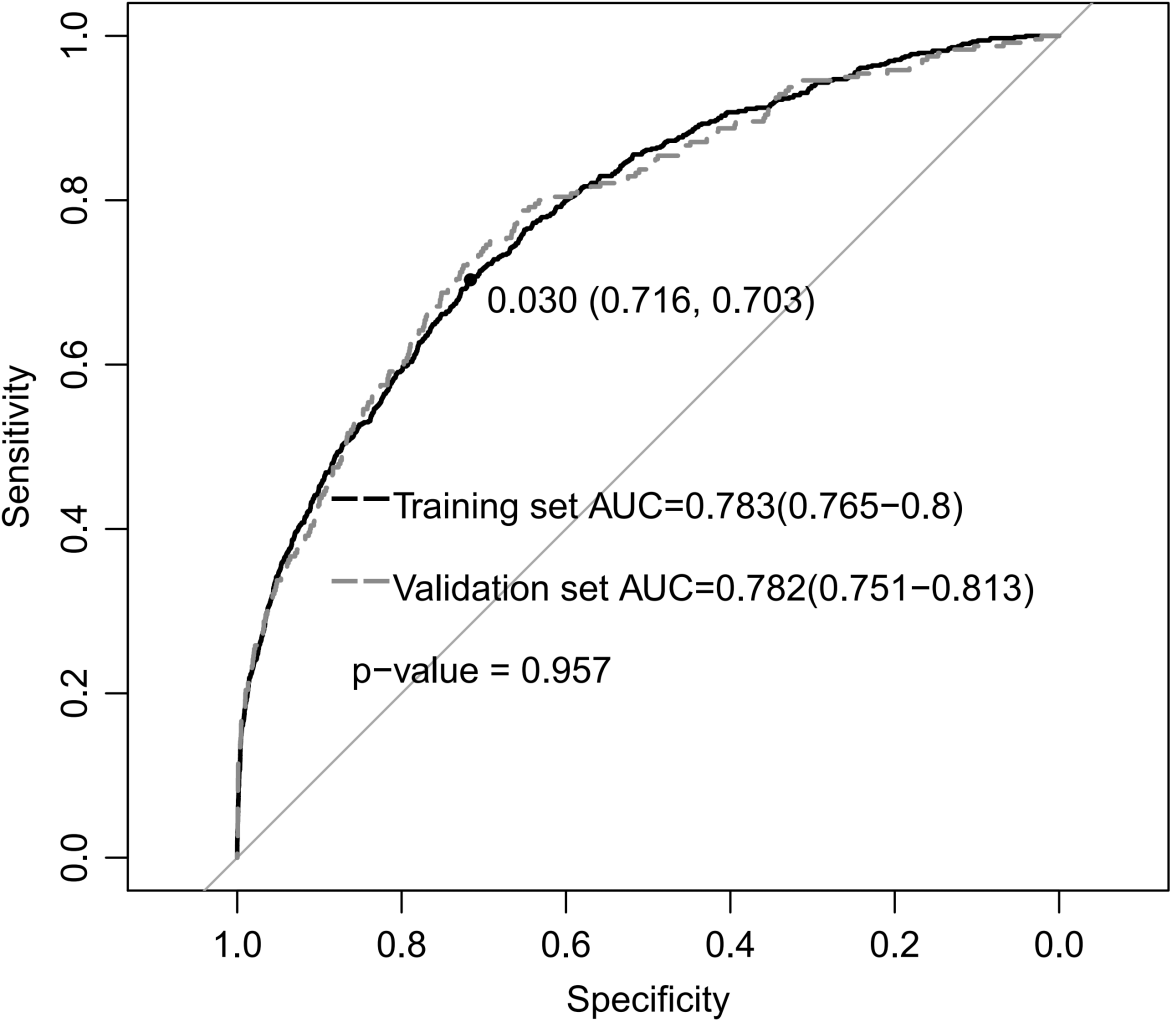
ROC curve in the training dataset and validation dataset

**FIGURE 2:**
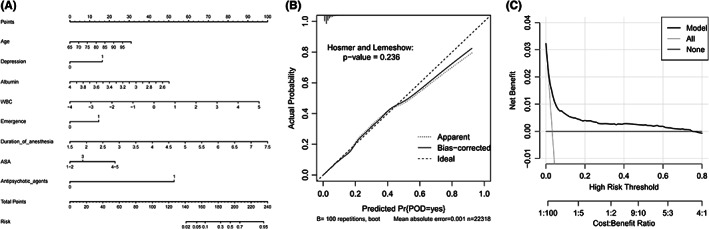
(A) Nomogram of logistic regression model. This nomogram was developed with eight perioperative predictors. Find each predictor's point on the uppermost point scale and add them up. The total point projected to the bottom scale indicates the % probability of POD. (B) Calibration curve of logistic regression model for the training dataset. (C) DCA of logistic regression model for the training dataset. ASA, American Society of Anesthesiologists physical status classification system; WBC, white blood cell. DCA, decision curve analysis

The remaining 7439 patients in the validation dataset of the logistic regression model were used to evaluate the performance of the predictive model. The prediction model had sufficient capacity with AUCs of 0.783 (0.765–0.8) and 0.782 (0.751–0.813) in the training and validation datasets (Figure 1). The accuracy, sensitivity, specificity, and precision were 70.9%, 74.2%, 70.7%, 7.8%, respectively (Table S4). Eight predictors were selected as optimal variables for predicting POD in the nomogram (Figure 2A). The interaction test was performed for the eight predictors. The ASA classification and use of antipsychotic agents had the most interaction with other variables (Table S2). So, we performed ROC for patients with ASA I‐III and without antipsychotic agents use, respectively. The AUC of patients with ASA I‐III is 0.775(0.743–0.807) (Figure S2A). The AUC of the dataset without antipsychotic agents use is 0.763(0.73–0.796) (Figure S2B). The prediction of the model also performed well in the two subgroups. Besides, the calibration curve showed good performance according to the Hosmer–Lemeshow test result (*p* = 0.086) (Figure 2B). DCA of the training dataset showed a satisfactory net benefit that the patient could receive from the predictive model. There was a wide range (5–75%) of high‐risk thresholds in the DCA (Figure 2C).

### 3.3 Development of different machine learning models for POD

Before constructing the machine learning models, the importance of all the variables was quantified and is shown in the chart in Figure S3 and Table S3. The WBC count, age, BMI, Glu level, and blood sodium level were the top five variables for predicting POD. The WBC count and age were also included in the logistic regression model. The normalized importance of the WBC count was 0.057 (range, 0–1), which was the highest of all variables. Therefore, it was difficult to predict POD using a small number of variables in machine learning models. The correlation of all the variables is also shown in the heatmap in Figure S4.

**FIGURE 3:**
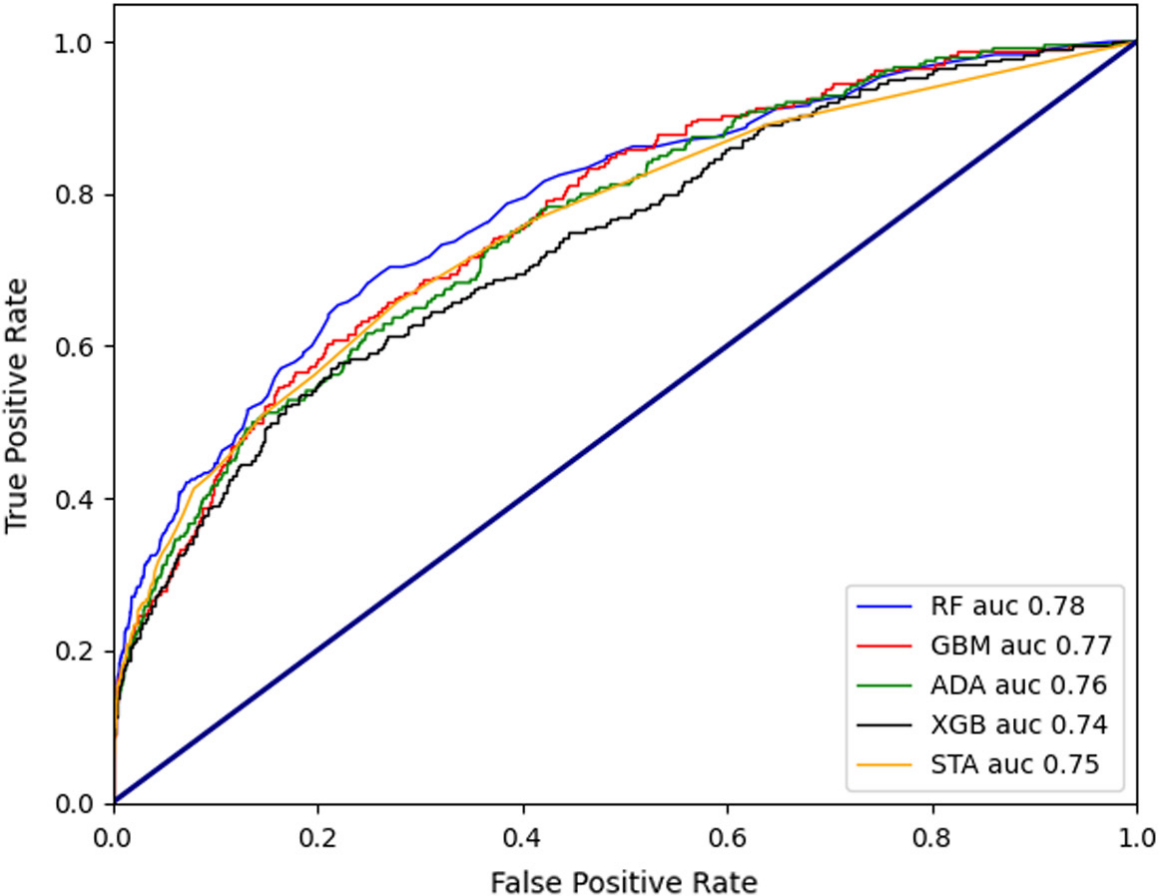
Comparison of ROCs and AUCs for prediction of POD by the various machine learning models. ROC, receiver operating characteristic curve; AUC, Area under the curve of ROC; POD, postoperative delirium. RF, random forest; GBM, gradient boosting algorithm; ADA, AdaBoost; XGB, XGBoost; STA, stacking ensemble model

The AUCs of the different machine learning models were as follows (Figure 3): 0.78 for RF, 0.76 for GBM, 0.74 for AdaBoost, 0.73 for XGBoost, and 0.77 for stacking ensemble model. The AUC of RF performed best.

The detailed results of the different machine learning models are shown in Table S4, which summarizes the statistics describing the models used to predict POD. These statistics include the AUC, which can be interpreted as the probability that a case would have a higher predicted value if presented with a random case and random control. Standard confusion matrix results were used to calculate the parameters of different models, including the accuracy (proportion of correct predictions in our model), sensitivity or recall (proportion of actual positives was identified correctly), specificity (proportion of actual negatives was identified correctly), precision (proportion of positive identifications was actually correct), and F1(2 × precision×recall/[precision+recall]). The accuracy ranged from 96.2% to 96.8% in the 5 models. GBM showed the best sensitivity up to 76.8%. RF showed the best specificity up to 99.8%. 57.0% was the best precision of the 5 models in AdaBoost. Stacking model indicated best F1(59.1%) (Table S4).

## 4 DISCUSSION

Previous studies have shown that most prediction models for POD focus on a particular surgical population^14^, ^30^. Mufti et al.^31^ developed POD prediction models of patients after cardiac surgery using logistic regression, artificial neural networks (ANN), support vector machines (SVM), Bayesian belief networks (BBN), naïve Bayesian, random forest, and decision trees. The best performance of the 7 prediction models was ANN with an AUC of 0.782. A POD prediction model of patients after microvascular decompression surgery achieved an AUC of 0.963 with RF algorithm^32^. In addition to the same surgery, the surgeon of microvascular decompression surgery is also the same individual in the dataset of 912 patients. Such prediction models usually perform well for the consistency of the population. This limitation is evident in that the sample size is usually not very large. Therefore, our model attempted to predict POD in a more general surgical population using a large dataset. Previous studies have shown that cardiac surgery and neurosurgery have a high incidence of POD, ranging from 16.2% to 32.4%^33^, ^34^. Early risk stratification and intervention have become the standard of care for such patients. Therefore, the development of an applicable prediction model for POD in patients undergoing non‐cardiac, non‐neurosurgical procedures is urgently needed.

We developed a logistic regression model and five machine learning models based on patients' perioperative data. The RF and logistic regression models achieved the same AUC (0.78). The results were almost same with Hu's work. They achieved best AUC of 0.8 with logistic regression algorithm in a dataset of 531 patients under general anesthesia^35^. Compared to small‐scale dataset, it is difficult to predict POD in a big dataset. Xue et al. constructed prediction models for 5 postoperative complications (acute kidney injury, delirium, deep vein thrombosis, pulmonary embolism, and pneumonia) with 5 machine learning algorithms (logistic regression, support vector machine, random forest, gradient boosting tree, and deep neural network) in a big dataset with 111,888 patients^19^. Compared to other postoperative complications, the AUC of delirium was lowest with 0.76.

We compared several machine learning models and found that the RF model had the best performance. The performance of machine learning models varies across studies^15^, ^20^, ^36^. Zhang et al.^37^ reported that the ensemble learning model had a good effect on predicting the agitation of patients in the intensive care unit (ICU) under light sedation. Therefore, we also attempted to use an ensemble learning model for prediction. The ensemble model did not show significant superiority in prediction after stacking the RF, AdaBoost, XGBoost, and GBM models. There are several possible reasons for no improvement of stacking model. First, the variables in the dataset did not show complicate relationships. Second, Adaboost and Xgboost are both ensemble learning methods. The RF is a classification algorithm consisting of many decisions trees. Gradient boosting is a machine learning technique used in regression and classification tasks, among others. It gives a prediction model in the form of an ensemble of weak prediction models. Most machine learning models have learned the complex relationship for all subtypes of patients, so further stacking did not provide additional improvement in model performance. To improve the performance of prediction, more methods needed to explore.

Compared with machine learning models, the logistic regression model showed better sensitivity and lower accuracy. The precision of the logistic regression was 7.8%, with a cutoff value of 0.03. The logistic regression model compromised precision to achieve a better AUC and sensitivity. After comprehensive consideration, we also chose the prediction model based on logistic regression analysis for three reasons. First, the incidence of POD was only 3.23%. The dataset was severely imbalanced. If one prediction model guessing all the patients without POD, it also can achieve a good accuracy[(TP + TN)/(TP + TN + FP + FN)]. So, the accuracy is not so crucial in comparing different models. Second, for patients with POD, sensitivity is more important than precision. Once patients develop POD, the medical costs and adverse outcomes will double and even more. Third, the logistic regression model achieved the same AUC with only eight variables, which are easier to use in combination with nomograms. All variables are interpretable and quantifiable, eliminating the “black box” in machine learning. This result is not surprising. Christodoulou et al.^21^ made a systematic review showing no performance benefit of machine learning over logistic regression for clinical prediction models.

This study has some limitations. First, it was a retrospective study. Only a retrospective dataset can provide a large sample size. Because assessing POD in a retrospective dataset using the confusion assessment method (CAM) or 3D‐CAM is not achievable, patients with POD were identified based on medical records using DSM‐IV criteria^2^, ^24^. We enrolled all patients undergoing surgery, including ophthalmic, ear–nose–throat, and oral surgery with a low POD incidence and excluded cardiac surgery and neurosurgery with a high POD incidence. Therefore, the 3.2% incidence of POD in our study is reasonable and consistent with that reported in previous studies^5^. Compared to patients with missing hypoactive POD, those with mixed and hyperactive POD recorded in medical records always need urgent intervention for their poor prognosis^38^. Therefore, developing prediction models for patients with POD is meaningful. Prospective research may provide more variables for prediction and more accurate assessment of POD. We are currently conducting a multicenter prospective study to identify POD patients using CAM, CAM‐ICU, and 3D‐CAM. Second, there was no external verification of the model. Therefore, extensive application of the model results may be limited. External validation will be performed in a multicenter prospective study. Finally, the model's performance in our study was only moderate, with an AUC <80%; thus, the effectiveness of using these prediction models is still open to debate. There are a lot of machine learning algorithms. The performance of different machine learning models varies across studies. But we only selected some commonly used algorithms to test. Besides the decision tree model (RF), ensemble learning methods (Adaboost and Xgboost), regression and classification method (Gradient boosting), we also tried the stacking model of above methods for POD prediction. But it did not show a better performance than other models. A bigger dataset of different clinical centers may provide more valuable information for prediction of POD. The transfer learning and federated learning also should be explored to achieve more ideal performance.

In summary, we constructed six prediction models for POD using logistic regression, RF, AdaBoost, XGBoost, GBM, and stacking ensemble learning based on retrospective analysis of a large sample dataset. The logistic regression model performed better than the machine learning models because of its better sensitivity, fewer variables, and easier interpretability. The optimal application of these models would provide quick and convenient POD risk stratification to help improve the perioperative management of general surgical patients.

## AUTHOR CONTRIBUTIONS

Wei‐dong Mi and Jiang‐bei Cao conceptualized the study. Jiang‐bei Cao and Yi‐qiang Chen designed the study. Yu‐xiang Song, Yun‐gen Luo, Chun‐lei Ouyang, and Yao Yu collected the data. Yu‐xiang Song, Xiao‐dong Yang analyzed and interpreted the data. Hao Li, Jing‐sheng Lou, Yu‐long Ma, and Yan‐hong Liu contributed for statistical analysis. Yu‐xiang Song and Xiao‐dong Yang drafted the manuscript. Wei‐dong Mi, Jiang‐bei Cao, Yi‐qiang Chen critically revised the manuscript. All authors gave the approval of final version of paper.

## FUNDING INFORMATION

This work was supported by a grant from the National Key Research and Development Program of China lead by Dr. Wei‐dong Mi (No.2018YFC2001901), which provided financial support for the study design, data collection and statistical analysis.

## CONFILCT OF INTEREST

None

## Supporting information


Appendix S1

## Data Availability

All the data shown in this study are available from the corresponding author upon request.
