# Supplementary material for: Comparison of logistic regression and machine learning methods for predicting postoperative delirium in elderly patients: A retrospective study
Source: CNS Neurosci Ther. 2022 Oct 11;29(1):158–67. doi: 10.1111/cns.13991 (PMC9804041; doi:10.1111/cns.13991)
Supplement: Supplementary file 1 — Appendix S1 [file CNS-29-158-s001.docx]

**
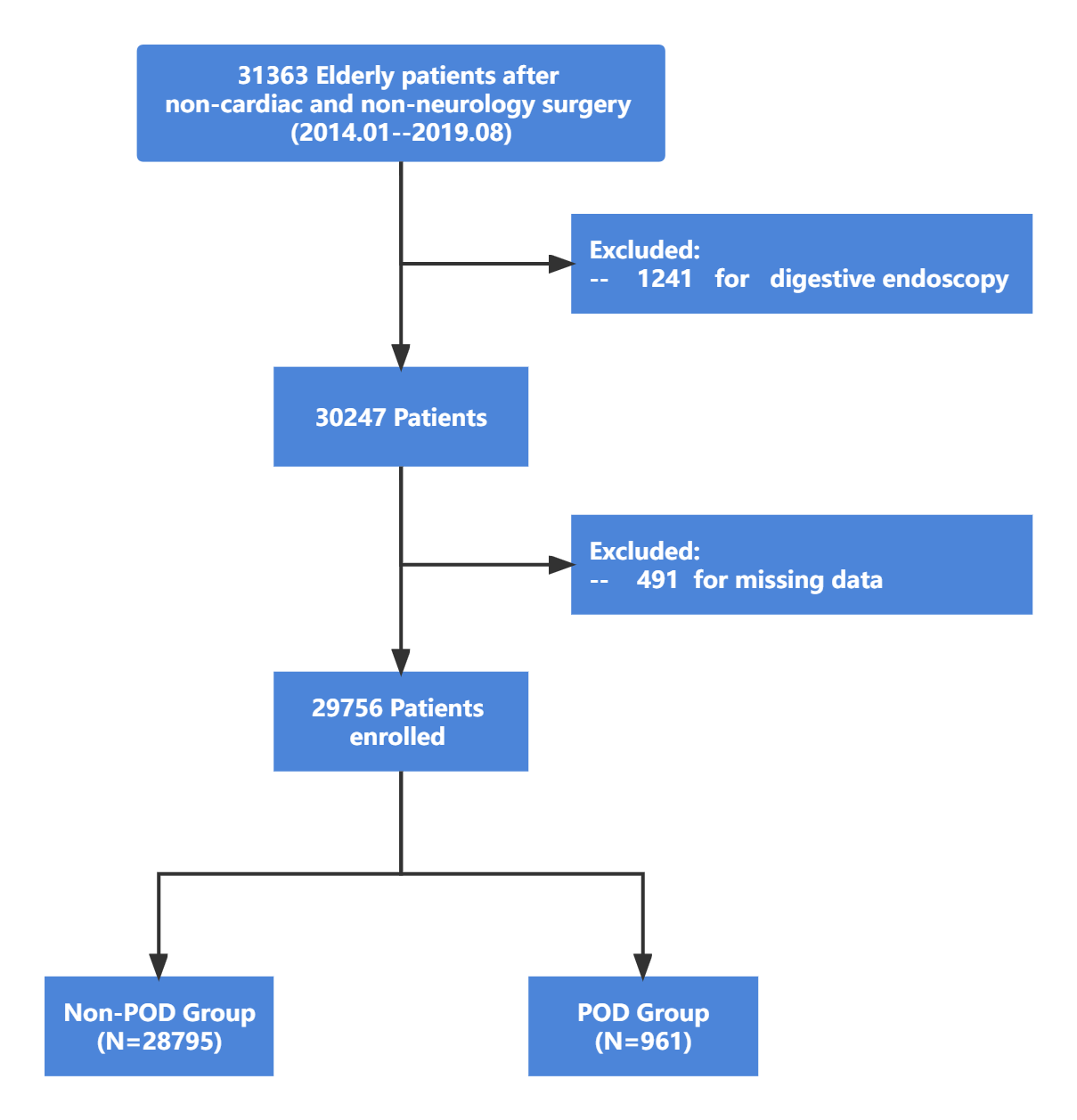
**

**Fig S1. The enrolment flow chart of patients.**

**Table S1 The univariate logistic regression analysis results of perioperative factors associated with POD. BMI, body mass index; COPD, chronic obstructive pulmonary disease; CKD, chronic kidney disease; ASA, American Society of Anesthesiologists physical status classification system; E.N.T, otolaryngology head and neck surgery; SBP, systolic blood pressure; MAP, mean arterial pressure; WBC, white blood cell; ESR, erythrocyte sedimentation rate; Glu, glucose; Cre, creatinine; AST, aspartate aminotransferase; ALT, alanine aminotransferase; NSAIDs, non-steroidal anti-inflammatory drugs**

| Variables | Odds Ratio (95%CI) | P-value |
| --- | --- | --- |
| Age, years | 1.092(1.079-1.105) | <0.001 |
| Sex, male vs female | 0.785(0.675-0.911) | 0.001 |
| BMI, kg·m-2 | 0.935(0.916-0.955) | <0.001 |
| Smoke, yes vs no | 1.219(1.029-1.438) | 0.02 |
| Alcohol, yes vs no | 1.136(0.951-1.349) | 0.153 |
| Hypertension, yes vs no | 1.124(0.969-1.304) | 0.123 |
| Diabetes, yes vs no | 1.178(0.994-1.391) | 0.056 |
| Cardiovascular diseases, yes vs no | 1.317(1.045-1.64) | 0.017 |
| COPD, yes vs no | 2.519(1.925-3.245) | <0.001 |
| Cerebrovascular disease, yes vs no | 1.658(1.341-2.033) | <0.001 |
| Parkinson’s disease, yes vs no | 4.335(2.239-7.661) | <0.001 |
| CKD, yes vs no | 3.001(1.911-4.497) | <0.001 |
| Depression, yes vs no | 4.292(2.28-7.427) | <0.001 |
| Non-independent functional status, yes vs no | 2.043(1.759-2.372) | <0.001 |
| ASA, vs I-II |  |  |
| III | 2.745(2.338-3.217) | <0.001 |
| IV-V | 15.896(11.304-22.045) | <0.001 |
| Emergency surgery, yes vs no | 4.563(3.542-5.806) | <0.001 |
| Type of surgery, vs Hepatopancreatobiliary and gastrointestinal surgery |  |  |
| Orthopedic surgery | 0.826(0.691-0.985) | 0.034 |
| Urinary surgery | 0.602(0.438-0.809) | 0.001 |
| Thoracic surgery | 0.527(0.364-0.74) | <0.001 |
| E.N.T | 0.442(0.274-0.673) | <0.001 |
| Vascular surgery | 1.034(0.722-1.44) | 0.847 |
| Stomatology | 0.529(0.324-0.813) | 0.006 |
| Gynecology | 0.703(0.436-1.074) | 0.124 |
| Thyroid and Brest | 0.485(0.256-0.83) | 0.015 |
| Anesthesia method, vs General anesthesia |  |  |
| Epidural anesthesia | 0.374(0.133-0.814) | 0.029 |
| General anesthesia combined other anesthesia | 0.972(0.767-1.217) | 0.812 |
| Nerve blocks | 1.422(0.883-2.162) | 0.122 |
| Basal anesthesia | 0.886(0.421-1.625) | 0.722 |
| Duration of surgery, min | 1.004(1.003-1.005) | <0.001 |
| Duration of anesthesia, min | 1.004(1.003-1.005) | <0.001 |
| Blood loss, ml | 1(1-1.001) | <0.001 |
| Urine, ml vs 0 |  |  |
| >0 and≤100 | 3.389(2.345-5.044) | <0.001 |
| >100 and ≤200 | 4.039(2.753-6.083) | <0.001 |
| >200 and ≤500 | 4.376(3.071-6.44) | <0.001 |
| >500 | 5.208(3.663-7.652) | <0.001 |
| Crystalloid, ml | 1(1-1) | <0.001 |
| Colloid, ml vs 0 |  |  |
| >0 and≤500 | 1.851(1.537-2.24) | <0.001 |
| >500 | 3.045(2.474-3.754) | <0.001 |
| Duration of SBP>140 mmHg, min vs 0 |  |  |
| >0 and≤10 | 1.125(0.909-1.389) | 0.276 |
| >10 | 1.586(1.335-1.89) | <0.001 |
| Duration of MAP<60 mmHg, min vs ≤10 |  |  |
| >10 and≤30 | 1.416(1.18-1.693) | <0.001 |
| >30 | 3.168(2.495-3.982) | <0.001 |
| Hemoglobin, g·L−1 | 0.973(0.969-0.977) | <0.001 |
| WBC count, *109/L | 1.148(1.122-1.175) | <0.001 |
| Glu, mmol/L | 1.156(1.123-1.19) | <0.001 |
| Serum albumin, g/L | 0.007(0.004-0.013) | <0.001 |
| Cre, μmol/L | 1.004(1.003-1.005) | <0.001 |
| Blood potassium, mmol/L | 1.097(0.888-1.352) | 0.388 |
| Blood sodium, mmol/L | 0.874(0.858-0.891) | <0.001 |
| Blood calcium, mmol/L | 0.093(0.055-0.158) | <0.001 |
| Total bilirubin, μmol/L | 1.004(1.002-1.005) | <0.001 |
| AST, U/L |  | 0.017 |
| ALT, U/L | 1(0.998-1.002) | 0.939 |
| Preoperative medication, yes vs no |  |  |
| Anticholinergic drug | 0.918(0.791-1.066) | 0.26 |
| NAISDs | 0.726(0.511-1) | 0.061 |
| Benzodiazepines | 1.085(0.908-1.289) | 0.363 |
| Opioids | 3.005(2.249-3.942) | <0.001 |
| Antipsychotic drugs | 63.385(39.997-102.771) | <0.001 |
| Intraoperative medication, yes vs no |  |  |
| Glucocorticoid | 1.019(0.875-1.19) | 0.806 |
| Dexmedetomidine | 1.193(0.94-1.495) | 0.136 |
| Droperidol | 0.794(0.591-1.043) | 0.11 |

**Table S2 The test of interaction of variables in the prediction model. ASA, American Society of Anesthesiologists physical status classification system; WBC, white blood cell.**

| **P for interaction** | **Age** | **Depression** | **Albumin** | **WBC** | **Emergence** | **Duration of anesthesia** | **ASA** |
| --- | --- | --- | --- | --- | --- | --- | --- |
| **Depression** | 0.48 | - | - | - | - | - | - |
| **Albumin** | 0.155 | 0.016 | - | - | - | - | - |
| **WBC** | 0.093 | 0.351 | 0.84 |  | - | - | - |
| **Emergence** | 0.239 | 0.115 | 0.211 | 0.837 | - | - | - |
| **Duration of anesthesia** | 0.272 | 0.697 | <0.01 | 0.062 | 0.63 | - | - |
| **ASA** | 0.016 | 0.474 | 0.112 | 0.405 | <0.01 | 0.042 | - |
| **Antipsychotic agents** | 0.011 | <0.01 | <0.01 | 0.07 | 0.962 | 0.204 | 0.041 |


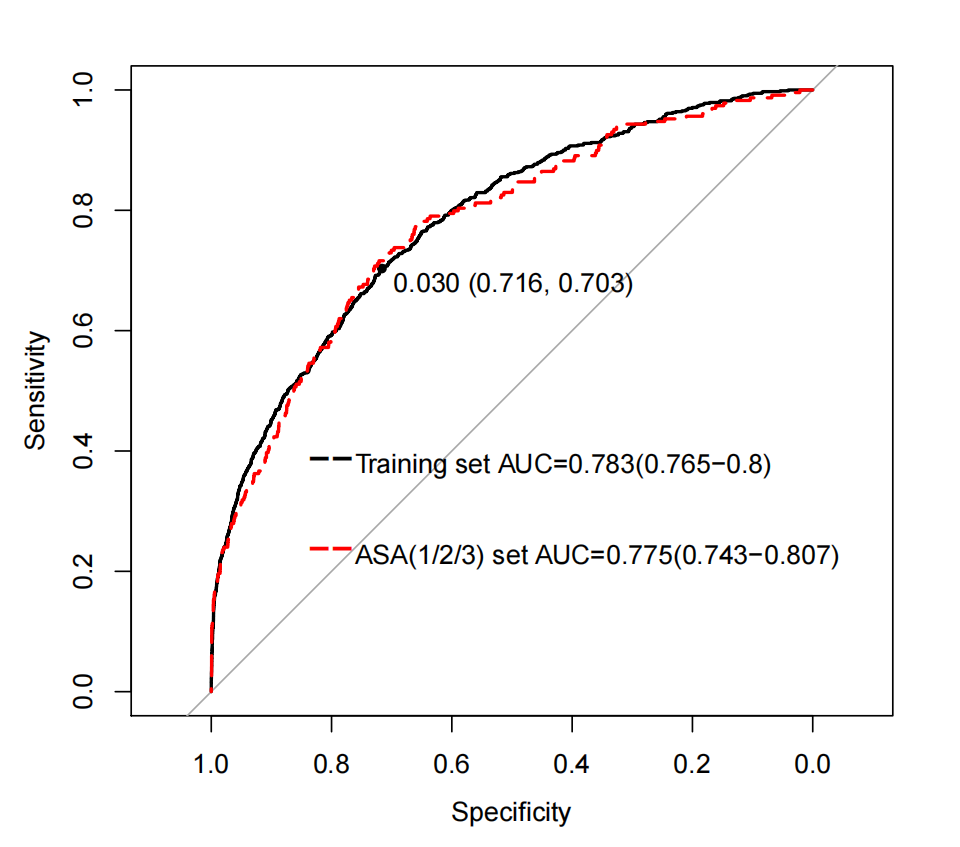


**Fig. S2a The AUC of patients with ASA I-III**


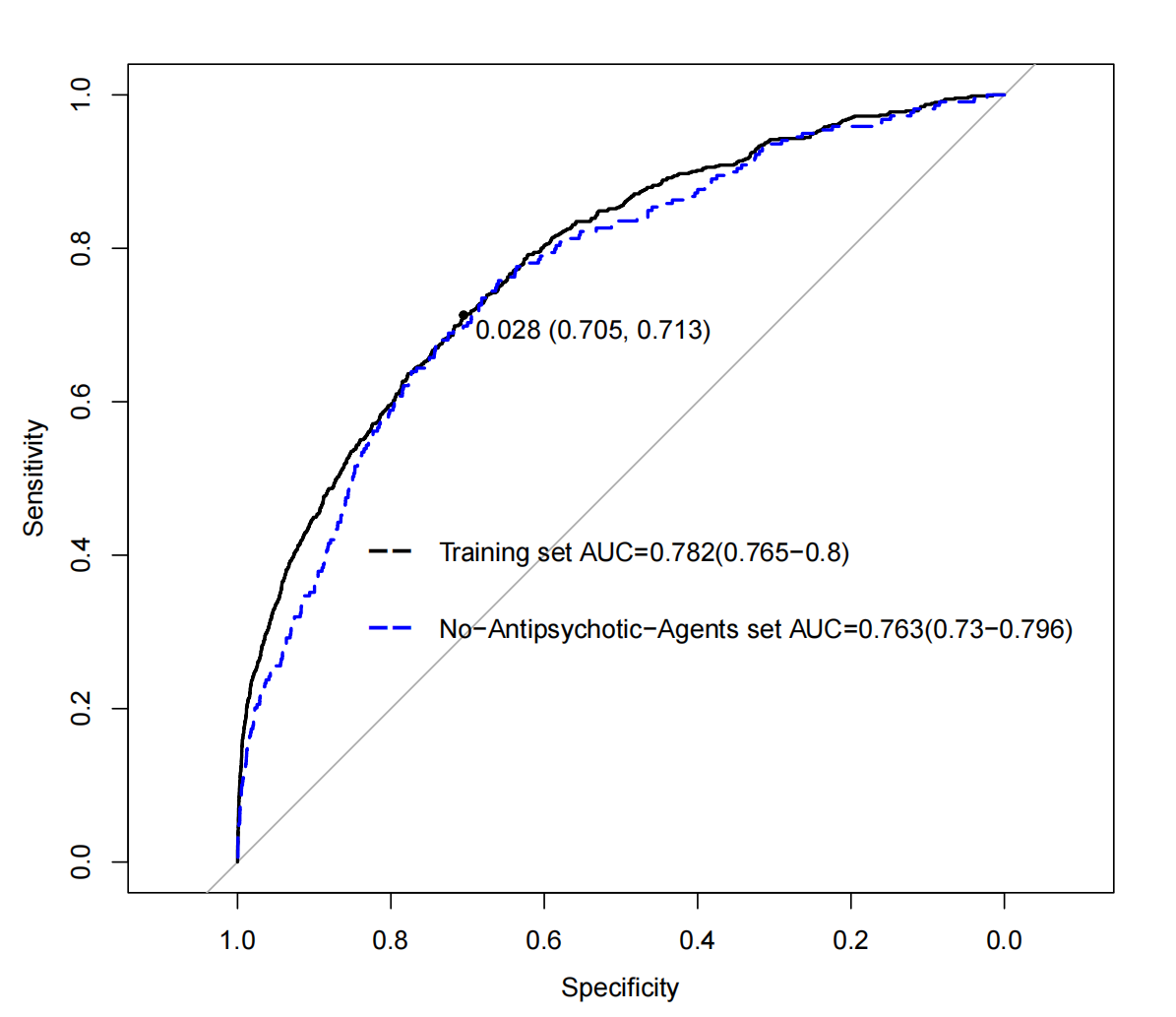


**Fig. S2b The AUC of patients without antipsychotic agents use**


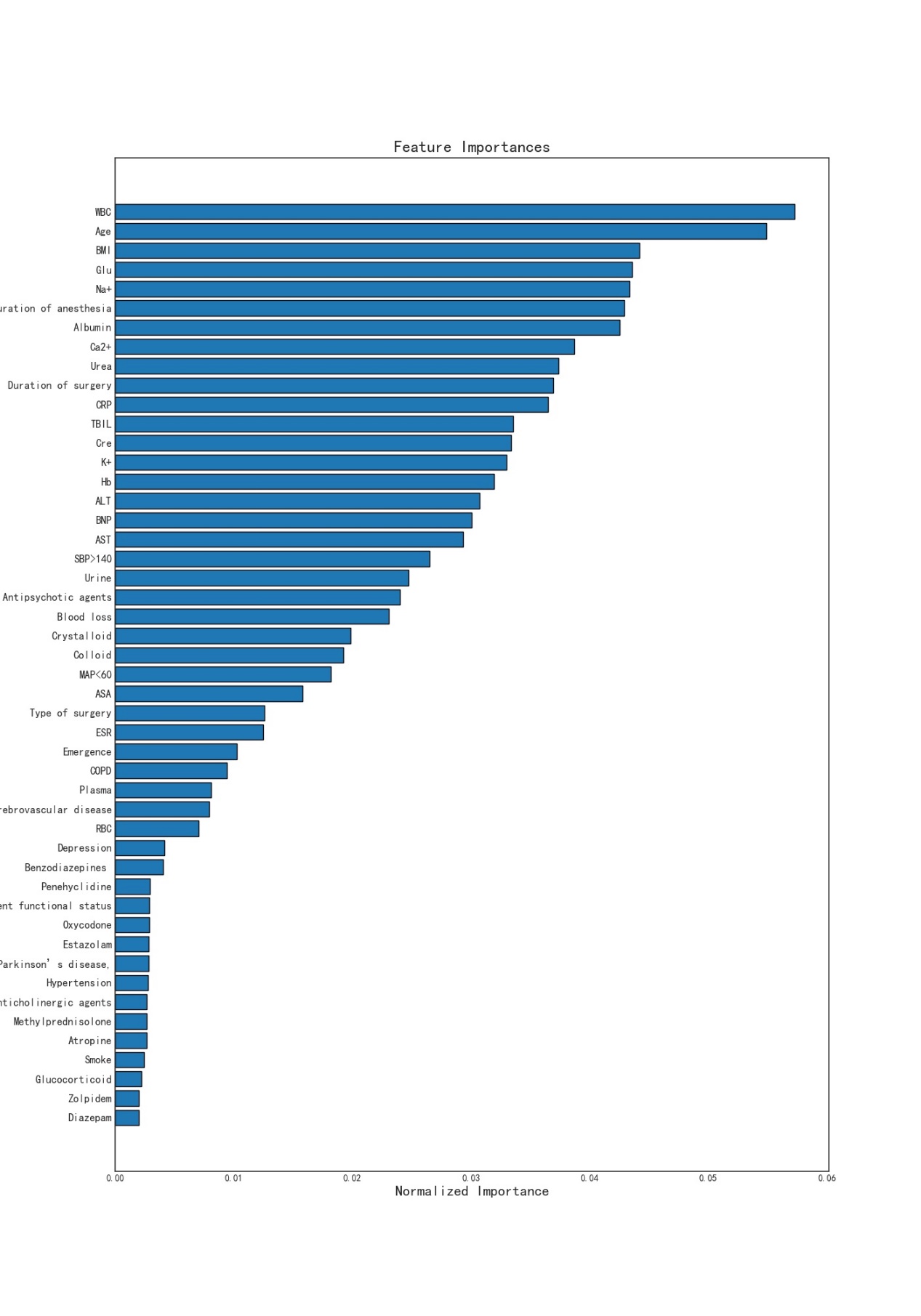


**Fig S3. The importance of all the variables. BMI, body mass index; COPD, chronic obstructive pulmonary disease; CKD, chronic kidney disease; ASA, American Society of Anesthesiologists physical status classification system; E.N.T, otolaryngology head and neck surgery; SBP, systolic blood pressure; MAP, mean arterial pressure; WBC, white blood cell; ESR, erythrocyte sedimentation rate; Glu, glucose; Cre, creatinine; AST, aspartate aminotransferase; ALT, alanine aminotransferase; NSAIDs, non-steroidal anti-inflammatory drugs**


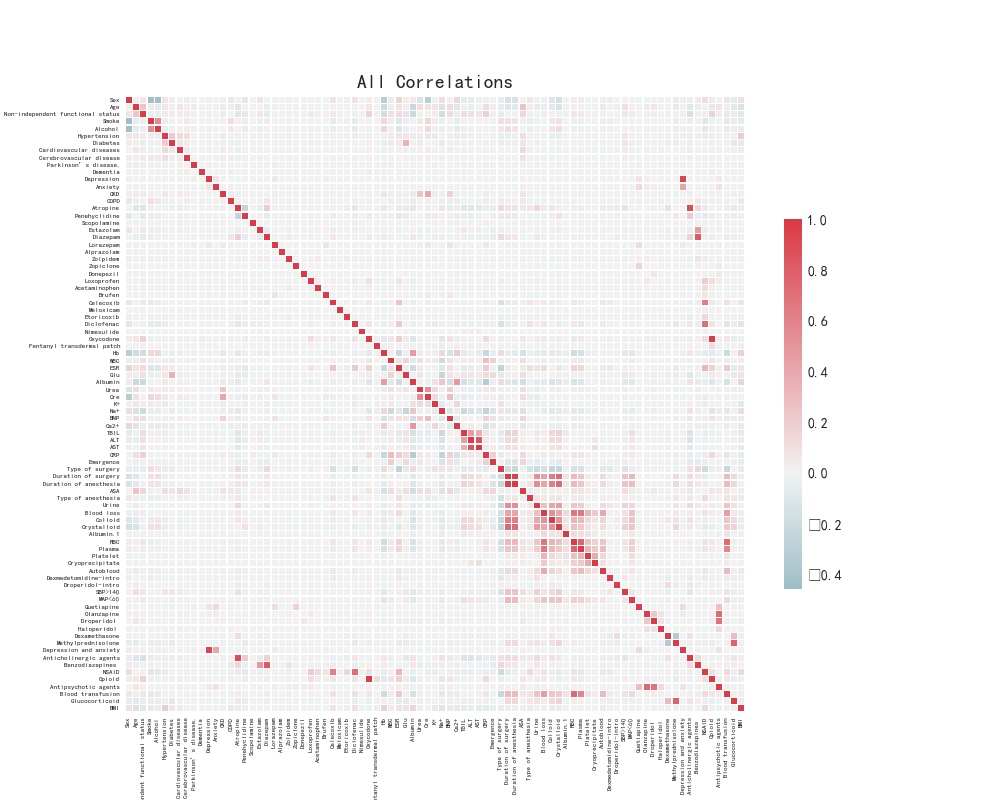


**Fig S4. The heatmap of all the variables correlation. The red and blue blocks show positive and negative correlations, respectively. BMI, body mass index; COPD, chronic obstructive pulmonary disease; CKD, chronic kidney disease; ASA, American Society of Anesthesiologists physical status classification system; E.N.T, otolaryngology head and neck surgery; SBP, systolic blood pressure; MAP, mean arterial pressure; WBC, white blood cell; ESR, erythrocyte sedimentation rate; Glu, glucose; Cre, creatinine; AST, aspartate aminotransferase; ALT, alanine aminotransferase; NSAIDs, non-steroidal anti-inflammatory drugs**

**Table S3 The exact data of importance of all the variables. BMI, body mass index; COPD, chronic obstructive pulmonary disease; CKD, chronic kidney disease; ASA, American Society of Anesthesiologists physical status classification system; E.N.T, otolaryngology head and neck surgery; SBP, systolic blood pressure; MAP, mean arterial pressure; WBC, white blood cell; ESR, erythrocyte sedimentation rate; Glu, glucose; Cre, creatinine; AST, aspartate aminotransferase; ALT, alanine aminotransferase; NSAIDs, non-steroidal anti-inflammatory drugs**

| **Variables** | **Importance** | **Normalized importance** |
| --- | --- | --- |
| **WBC** | 91.1 | 0.057295597 |
| **Age** | 87.3 | 0.05490566 |
| **BMI** | 70.3 | 0.044213836 |
| **Glu** | 69.3 | 0.043584906 |
| **Na+** | 68.9 | 0.043333333 |
| **Duration of anesthesia** | 68.2 | 0.042893082 |
| **Albumin** | 67.6 | 0.042515723 |
| **Ca2+** | 61.5 | 0.038679245 |
| **Urea** | 59.4 | 0.037358491 |
| **Duration of surgery** | 58.7 | 0.036918239 |
| **CRP** | 58 | 0.036477987 |
| **TBIL** | 53.3 | 0.033522013 |
| **Cre** | 53.1 | 0.033396226 |
| **K+** | 52.5 | 0.033018868 |
| **Hb** | 50.8 | 0.031949686 |
| **ALT** | 48.8 | 0.030691824 |
| **BNP** | 47.8 | 0.030062893 |
| **AST** | 46.6 | 0.029308176 |
| **SBP>140** | 42.1 | 0.026477987 |
| **Urine** | 39.3 | 0.024716981 |
| **Antipsychotic agents** | 38.2 | 0.024025157 |
| **Blood loss** | 36.7 | 0.023081761 |
| **Crystalloid** | 31.6 | 0.019874214 |
| **Colloid** | 30.6 | 0.019245283 |
| **MAP<60** | 28.9 | 0.018176101 |
| **ASA** | 25.1 | 0.015786164 |
| **Type of surgery** | 20 | 0.012578616 |
| **ESR** | 19.8 | 0.01245283 |
| **Emergence** | 16.3 | 0.010251572 |
| **COPD** | 15 | 0.009433962 |
| **Plasma** | 12.9 | 0.008113208 |
| **Cerebrovascular disease** | 12.6 | 0.007924528 |
| **RBC** | 11.2 | 0.007044025 |
| **Depression** | 6.6 | 0.004150943 |
| **Benzodiazepines** | 6.4 | 0.004025157 |
| **Penehyclidine** | 4.7 | 0.002955975 |
| **Non-independent functional status** | 4.6 | 0.002893082 |
| **Oxycodone** | 4.6 | 0.002893082 |
| **Estazolam** | 4.5 | 0.002830189 |
| **Parkinson’s disease,** | 4.5 | 0.002830189 |
| **Hypertension** | 4.4 | 0.002767296 |
| **Anticholinergic agents** | 4.2 | 0.002641509 |
| **Methylprednisolone** | 4.2 | 0.002641509 |
| **Atropine** | 4.2 | 0.002641509 |
| **Smoke** | 3.9 | 0.00245283 |
| **Glucocorticoid** | 3.5 | 0.002201258 |
| **Zolpidem** | 3.2 | 0.002012579 |
| **Diazepam** | 3.2 | 0.002012579 |

**Table S4 Comparison the parameters of models for prediction of POD**

|  | AUC | Accuracy | Sensitivity (Recall) | Specificity | Precision | F1 |
| --- | --- | --- | --- | --- | --- | --- |
| RF | 0.78 | 96.8% | 72.2% | 99.8% | 52.3% | 53.4% |
| GBM | 0.76 | 96.9% | 76.8% | 96.5% | 55.6% | 58.7% |
| AdaBoost | 0.74 | 96.2% | 63.6% | 98.8% | 57.0% | 59.0% |
| XGBoost | 0.73 | 96.8% | 71.6% | 96.5% | 54.5% | 57.0% |
| Stacking | 0.77 | 96.5% | 67.4% | 96.1% | 56.4% | 59.1% |
| LR | 0.78 | 70.9% | 74.2% | 70.7% | 7.8% | 14.1% |

AUC, area under the curve of ROC; RF, random forest; GBM, Gradient Boosting Machine; AdaBoost, Adaptive Boosting; XGBoost, eXtreme Gradient Boosting; Stacking, Stacking Ensemble model; LR, logistic regression;

Accuracy=(TP+TN)/(TP+TN+FP+FN)

Sensitivity = TP/ (TP + FN)

Specificity (Recall) = TN/ (TN + FP)

Precision=TP / (TP + FP)

F1=2*Precision*Recall/ (Precision + Recall)

TP = true positive

TN = true negative

FP = false positive

FN = false negative
